# Vitamin D Analogs Regulate the Vitamin D System and Cell Viability in Ovarian Cancer Cells

**DOI:** 10.3390/ijms23010172

**Published:** 2021-12-24

**Authors:** Karina Piatek, Andrzej Kutner, Dan Cacsire Castillo-Tong, Teresa Manhardt, Nadja Kupper, Urszula Nowak, Michał Chodyński, Ewa Marcinkowska, Enikö Kallay, Martin Schepelmann

**Affiliations:** 1 Institute for Pathophysiology and Allergy Research, Medical University of Vienna, Waehringer Guertel 18-20, 1090 Vienna, Austria; karina.piatek@meduniwien.ac.at (K.P.); teresa.manhardt@meduniwien.ac.at (T.M.); nadja.kupper@meduniwien.ac.at (N.K.); enikoe.kallay@meduniwien.ac.at (E.K.); 2 Department of Bioanalysis and Drug Analysis, Faculty of Pharmacy, Medical University of Warsaw, 1 Banacha, 02-097 Warsaw, Poland; andrzej.kutner@wum.edu.pl; 3 Translational Gynecology Group, Department of Obstetrics and Gynecology, Comprehensive Cancer Center, Medical University of Vienna, 1090 Vienna, Austria; dan.cacsire-castillo@meduniwien.ac.at; 4 Faculty of Biotechnology, University of Wrocław, Joliot-Curie 14a, 50-383 Wrocław, Poland; urszula.nowak2@uwr.edu.pl (U.N.); ema@cs.uni.wroc.pl (E.M.); 5 Łukasiewicz Research Network—Industrial Chemistry Institute, 8 Rydygiera, 01-793 Warsaw, Poland; michal.chodynski@ichp.pl

**Keywords:** vitamin D, vitamin D analogs, 25 vitamin D 24-hydroxylase, CYP24A1, proliferation, high-grade serous ovarian cancer cells

## Abstract

Background: Ovarian cancer (OC) is one of the most lethal cancers in women. The active form of vitamin D_3_, 1,25-dihydroxyvitamin D_3_ (1,25D_3_, calcitriol) has anticancer activity in several cancers, including ovarian cancer, but the required pharmacological doses may cause hypercalcemia. We hypothesized that newly developed, low calcemic, vitamin D analogs (an1,25Ds) may be used as anticancer agents instead of calcitriol in ovarian cancer cells. Methods: We used two patient-derived high-grade serous ovarian cancer (HGSOC) cell lines with low (13781) and high (14433) mRNA expression levels of the gene encoding 1,25-dihydroxyvitamin D_3_ 24-hydroxylase *CYP24A1*, one of the main target genes of calcitriol. We tested the effect of calcitriol and four structurally related series of an1,25Ds (PRI-1906, PRI-1907, PRI-5201, PRI-5202) on cell number, viability, the expression of *CYP24A1*, and the vitamin D receptor (VDR). Results: *CYP24A1* mRNA expression increased in a concentration-dependent manner after treatment with all compounds. In both cell lines, after 4 h, PRI-5202 was the most potent analog (in 13781 cells: EC_50_ = 2.98 ± 1.10 nmol/L, in 14433 cells: EC_50_ = 0.92 ± 0.20 nmol/L), while PRI-1907 was the least active one (in 13781 cells: EC_50_ = n/d, in 14433 cells: EC_50_ = n/d). This difference among the analogs disappeared after 5 days of treatment. The 13781 cells were more sensitive to the an1,25Ds compared with 14433 cells. The an1,25Ds increased nuclear VDR levels and reduced cell viability, but only in the 13781 cell line. Conclusions: The an1,25Ds had different potencies in the HGSOC cell lines and their efficacy in increasing *CYP24A1* expression was cell line- and chemical structure-dependent. Therefore, choosing sensitive cancer cell lines and further optimization of the analogs’ structure might lead to new treatment options against ovarian cancer.

## 1. Introduction

Ovarian cancer (OC), known as the “silent killer” showing no symptoms at early stages, is the deadliest gynecologic malignancy. Relapse after initial treatment is frequently fatal. Preventing relapse of the tumor is the ultimate, yet still unmet goal in treating OC patients. The standard treatment of OC is cytoreductive surgery and platinum-taxane-based chemotherapy. The use of platinum-based therapeutics is, however, limited in dosage and frequency due to their unspecific mode of action and toxic effects on normal cells, severely reducing the patients’ quality of life [1]. Very often, a small number of cells in the primary tumors escape the toxicity of the drugs. These cells will grow into a life-threatening recurrent disease that finally leads to death [1,2]. Low vitamin D levels were associated with increased risk for various cancers, suggesting a beneficial effect of supplementation or treatment with vitamin D, its active metabolites, or synthetic analogs [3,4].

Vitamin D_3_ is produced in the skin by sunlight from 7-dehydrocholesterol [5]. Cytochrome P450 enzymes activate vitamin D in two steps: 25-hydroxylation in the liver, followed by 1α-hydroxylation in the kidneys [6]. The most active form of vitamin D_3_, 1,25-dihydroxyvitamin D_3_ (calcitriol) binds to the vitamin D receptor (VDR), which acts as a ligand-activated transcription factor, binding to vitamin D response elements (VDRE) on target genes [7]. Liganded VDR upregulates the transcription of hundreds of target genes. The gene encoding 25-hydroxyvitamin D 24-hydroxylase (*CYP24A1*) is the most strongly upregulated one [8]. Hydroxylation of calcitriol at C-24, catalyzed by CYP24A1, initiates its catabolism to the inactive and water-soluble ultimate metabolite, calcitroic acid. CYP24A1 thus provides negative feedback to the activity of calcitriol [9].

The expression of *CYP24A1* is usually low in the absence of calcitriol [10]. The gene contains multiple *VDRE* sequences and is highly responsive to liganded VDR [8]. CYP24A1 is present in the inner mitochondrial membrane of all calcitriol responsive cells. After exposure to calcitriol, the expression of *CYP24A1* increases, however, the kinetics of the increase is cell-type specific [11]. Interestingly, several tumors have a high constitutive expression of *CYP24A1,* which may lower the local concentration of calcitriol in the cancer microenvironment [12].

The best-known role of calcitriol is to maintain the calcium-phosphate homeostasis of the organism [13]. It also stimulates other vital processes, such as the differentiation of keratinocytes, the development of immune cells, and the detoxification of the organism [14,15]. In numerous cancer cells, calcitriol inhibits proliferation and induces differentiation or apoptosis [4,16]. Different analogs of calcitriol (an1,25Ds; for simplicity, 1,25D includes both 1,25D_2_ and 1,25D_3_) were also shown to inhibit proliferation, modulate the immune response of different cancer cells, and reduce tumor growth and metastasis in animal models of various cancers [4,17].

Epidemiological data suggest that persons with low serum levels of the circulating form of vitamin D (25-hydroxyvitamin D, 25D), are at higher risk to develop HGSOC, and therefore, vitamin D supplementation might prevent ovarian carcinogenesis [18]. However, the use of calcitriol in therapeutic, supra-physiological doses is limited by its potent calcemic and phosphatemic activities. Over the years, several an1,25Ds were synthesized [19] with reduced or negligible calcemic and enhanced anticancer activity, as tested in leukemia, breast, prostate, and colon cancer cells [20,21,22,23,24]. However, an1,25Ds have not been extensively studied on OC cells, except for a single study of the 1,25D_3_ analog (EB1089) several years ago [25]. Thus, although there are some reports on the activity of calcitriol in commercially available ovarian cancer cells, little is known on the effects of an1,25Ds on HGSOC cells.

**Figure 1:**
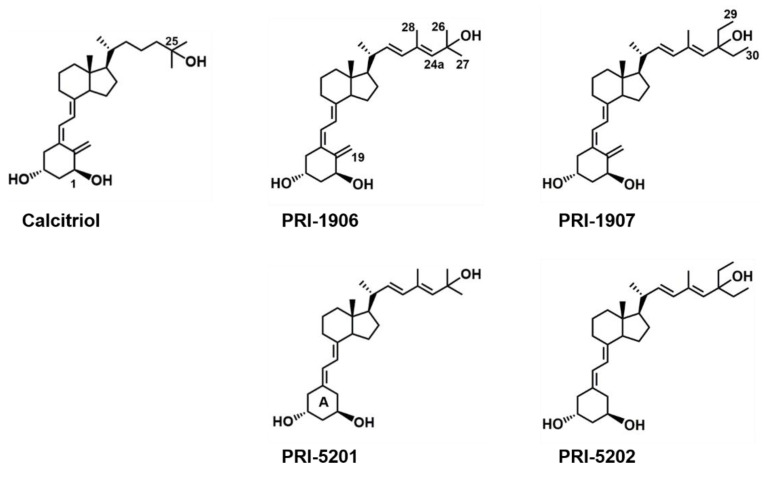
Chemical structures of calcitriol and the tested vitamin D analogs PRI-1906, PRI-1907, PRI-5201, and PRI-5202. Some carbons and rings are specifically labeled due to their structural importance (see discussion section).

We have previously shown that analogs of 1,25-dihydroxyergocalciferol (1,25-dihydroxyvitamin D_2_, 1,25D_2_) induced differentiation of human acute myeloid leukemia cells [21,26]. As the effect of calcitriol and of different an1,25Ds is very often cell- and tissue-dependent, in this study we tested if these novel, less calcemic an1,25Ds, known to be effective in colorectal and breast cancer models, are also effective in ovarian cancer cells. We compared the effect of a series of structurally related an1,25Ds (PRI-1906, PRI-1907, PRI-5201, PRI-5202, Figure 1) with that of calcitriol in two patient-derived HGSOC cell lines, to investigate if any of these analogs affect ovarian cancer cells and thus to evaluate the possibility of developing an1,25Ds as potential adjuvants for treatment of HGSOC.

## 2. Results

Synthetic an1,25Ds of the most active forms of vitamin D (1,25D_3_ and 1,25D_2_) have been tested against several types of cancer [4], however, very little is known about their activity in ovarian cancer cells. We have chosen two cell lines (13781 and 14433) from our previously established patient-derived HGSOC cell line panel [27] and treated them with either calcitriol, as a widely used reference, or four side chain-modified an1,25Ds (PRI-1906, PRI-1907, PRI-5201, and PRI-5202). The side-chain structure and especially the C-26, C-27 alkyls, contribute significantly to the binding affinity of the analog for VDR and thus to its functional activity. They surround the terminal C-25 hydroxyl group and affect the formation of a hydrogen bond with the amino acid residues His397 and His 397 of the ligand-binding pocket of the ligand-binding domain (LBD) of VDR [28].

### 2.1. Basal Expression of CYP24A1

The main known role of CYP24A1 (the 1,25-dihydroxy vitamin D 24-hydroxylase) enzyme, a mixed-function oxidase cytochrome P450 molecule, is to catabolize both the active form of vitamin D 1,25D and its precursor 25D, in order to prevent hypercalcemia.

**Figure 2:**
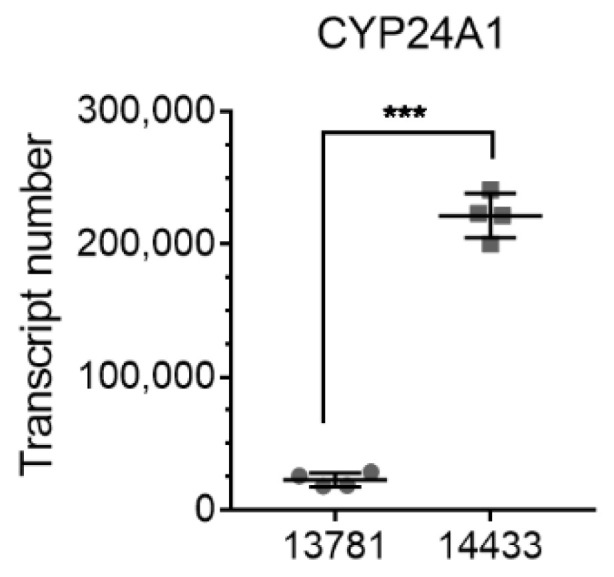
Absolute transcript number of CYP24A1 mRNA in the two cell lines 13781 and 14433 measured by absolute quantification RT-qPCR. Two-tailed *t*-test *** *p* < 0.001, N = 4.

Our previous RNA-seq data analysis [27] had already indicated that the 13781 cells have a lower basal level of CYP24A1 mRNA than 14433 cells. To validate the RNA-seq data, we measured the transcript number (Q) of the *CYP24A1* gene using quantitative real-time PCR (RT-qPCR). The results confirmed the data from the RNA-seq analysis. Basal level of CYP24A1 is around 10 times higher in 14433 cells (Q = 221,477 ± 16,629) than in 13781 cells (Q = 22,642 ± 5351) (Figure 2).

### 2.2. Effect of Treatment with an1,25Ds on CYP24A1 Expression

As *CYP24A1* is one of the best-known targets most strongly affected by activation of VDR, we have used its expression as a marker for the activity of the an1,25Ds in the treated cells.

#### 2.2.1. Effect of Short-Term Treatment with an1,25Ds on *CYP24A1* Expression

**Figure 3:**
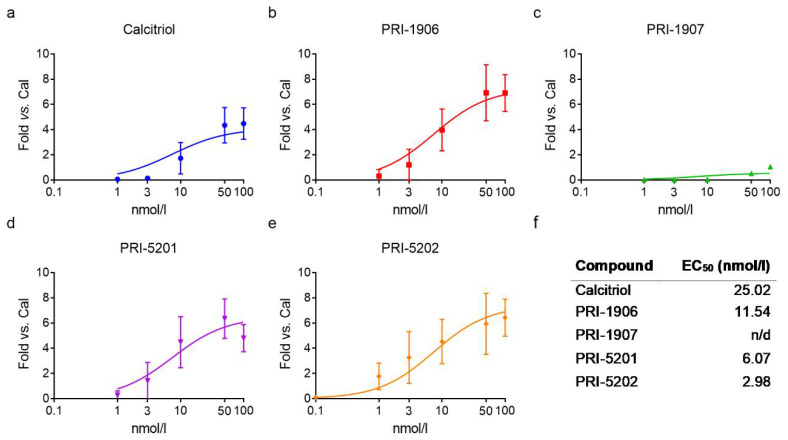
Activity of the compounds in the 13781 cell line. (**a**–**e**) Concentration-response curves of *CYP24A1* expression after treatment with the an1,25Ds for 4 h. Mean ± SD, nonlinear regression: sigmoidal three parameter fit with the following constraints: bottom > 0, EC_50_ > 0; N = 3–8. (**f**) The table shows the calculated EC_50_ values (n/d = not determined).

**Figure 4:**
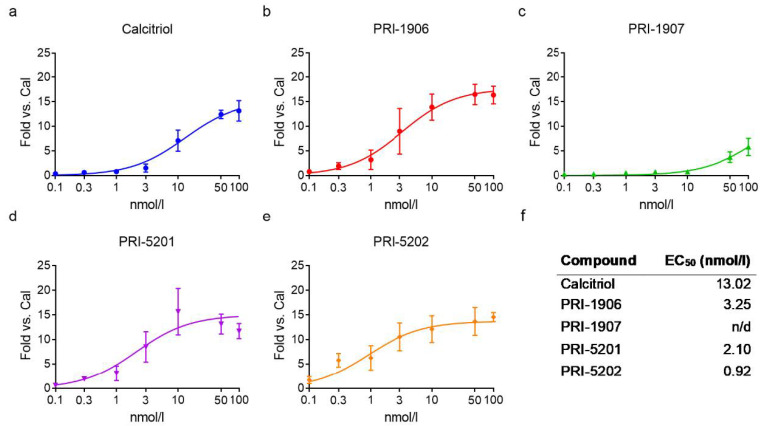
Activity of the compounds in the 14433 cell line. (**a**–**e**) Concentration-response curves for *CYP24A1* expression after treatment with the an1,25Ds for 4 h. Mean ± SD, nonlinear regression: sigmoidal three parameter fit with the following constraints: bottom > 0, EC_50_ > 0; N = 3–12. (**f**) The table shows the calculated EC_50_ values (n/d = not determined).

We treated the cells with the an1,25Ds at different concentrations (i.e., 0.1; 0.3; 1; 3; 10; 50; 100 nmol/L) for 4 h and calculated the EC_50_ (half-maximal effective concentration) for their effect on *CYP24A1* gene induction (Figure 3 and Figure 4). In both cell lines, PRI-5202 was the most active analog with the lowest EC_50_, and PRI-1907 was the least active one. The potency of the analogs was around two times higher in the 14433 cell line, which has a higher *CYP24A1* basal expression level than in the 13781 cell line.

#### 2.2.2. Effect of Long-Term Treatment with an1,25Ds on Gene Expression

**Figure 5:**
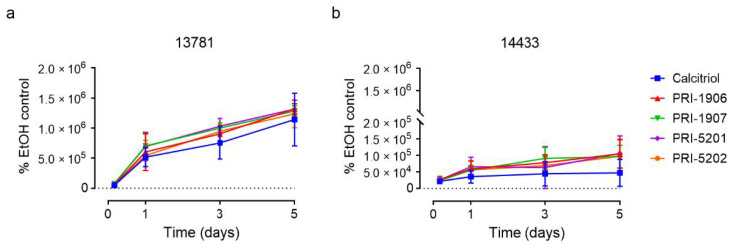
Expression of *CYP24A1* in (**a**) 13781 and (**b**) 14433 cells after treatment with an1,25Ds at 100 nmol/L at different time points (4 h, 1 day, 3, and 5 days). The diagrams show the percentage increase compared with the ethanol control. N = 3.

To study the long-term effect of the an1,25Ds, the cells were treated with each compound at 100 nmol/L for 4 h, 1, 3, and 5 days. After 5 days of treatment, the effect was stronger in the 13781 cells (Figure 5a) than in the 14433 cells (Figure 5b). This outcome probably results from the already high basal levels of *CYP24A1* mRNA in the 14433 cells, where an expression plateau is reached earlier.

When we compared the effectiveness of the an1,25Ds in the two cell lines, we realized that the increase in *CYP24A1* expression compared to the basal level was always higher in the 13781 cells, compared with the 14433 cells (Table 1, raw values with SD used to calculate these ratios are shown in Supplementary Tables S1 and S2). While we see a stronger effect of PRI-5202 at 1 nmol/L, this difference disappears at a high concentration (100 nmol/L).

We also tested the effect of an1,25Ds on other genes connected with inflammation and tumorigenesis (Supplementary Figure S1). In the 13781 cells, we observed that at a concentration of 100 nmol/L, all analogs increased the expression of CXCL1, CXCL2, IL-6, and IL-8 after 4 h after which the expression levels returned to the levels of the solvent control, On the other hand, the 14433 cells reacted with initial downregulation of CXCL1 and CXCL2 before returning to baseline levels while IL-6 and IL-8 were not affected by the treatments at all. These results again demonstrated the differential responsiveness of these cells to an1,25Ds.

### 2.3. Effect of Treatment with an1,25Ds on Nuclear VDR Level

**Figure 6:**
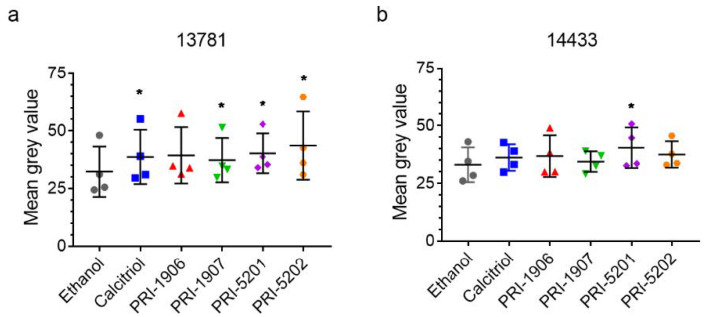
Nuclear VDR protein level in (**a**) 13781 and (**b**) 14433 cells after treatment with 100 nmol/L of an1,25Ds for 5 days. RM-ANOVA with Dunnett post-test vs. ethanol control, * *p* < 0.05, N = 4.

To induce gene expression, the an1,25Ds bind to VDR, which is a transcription factor. In some cells, the ligands of VDR can either upregulate its expression or stabilize the protein. Therefore, our next step was to study the effect of the an1,25Ds on VDR protein level in the nucleus. The cells were treated for 5 days with each compound at a concentration of 100 nmol/L. After the treatment, we stained the cells and analyzed the VDR staining intensity in the nuclei of the cells using single-cell quantitative immunofluorescence. In the 13781 cell line, all compounds except PRI-1906 increased the nuclear intensity for VDR, indicating higher nuclear VDR protein levels (Figure 6a, Supplementary Table S3). In the 14433 cell line, only PRI-5201 increased nuclear VDR protein levels (Figure 6b, Supplementary Table S3).

### 2.4. Effect of Treatment with an1,25Ds on the Proliferation of Ovarian Cancer Cells

As our previous experiments showed that the compounds affect the vitamin D system of the cells, we investigated the effect of the compounds on different markers of proliferation.

#### 2.4.1. Effect of Treatment with an1,25Ds on Cell Number and Viability

**Figure 7:**
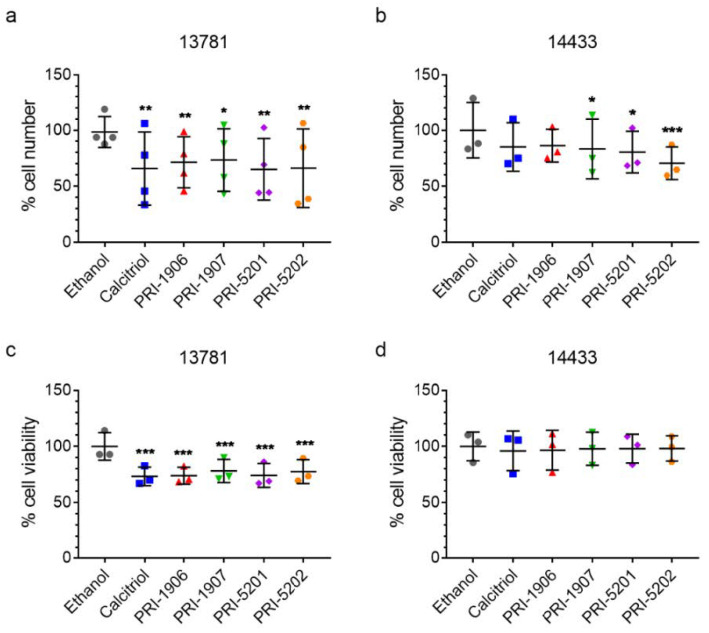
Cell number and viability in (**a**,**c**) 13781, and (**b**,**d**) 14433 cells after treatment with an1,25Ds at a concentration of 100 nmol/L for 5 days. RM-ANOVA with Dunnett post-test vs. ethanol control, * *p* < 0.05, ** *p* < 0.01, *** *p* < 0.01, N = 4 (**a**), 3 (**b**–**d**).

In the 13781 cell line, all tested compounds significantly reduced the cell number (Figure 7a). In contrast, in the 14433 cell line a statistically significant reduction in the number of cells was observed only after treatment with PRI-1907, PRI-5201, and PRI-5202, but not with calcitriol and PRI-1906 (Figure 7b). In addition to cell number, we measured cell viability directly. In the 13781 cell line, all tested compounds significantly reduced the viability of the cells (Figure 7c). In the 14433 cell line, we observed no significant effects on cell viability (Figure 7d), again highlighting the differences between the individual cell lines.

#### 2.4.2. Effect of Treatment with an1,25Ds on Ki67 Expression

**Figure 8:**
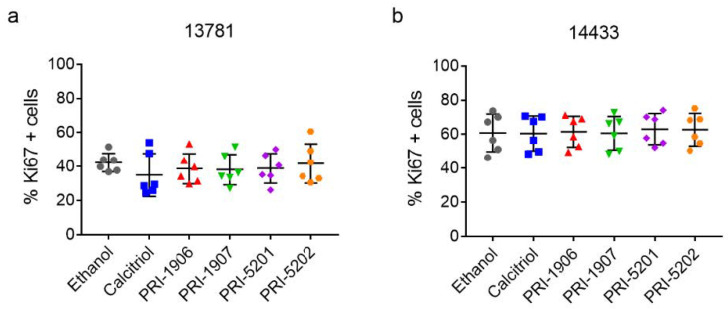
Percentage of KI67 positive cells in (**a**) 13781 and (**b**) 14433 cells after treatment with 100 nmol/L of an1,25Ds for 5 days. RM-ANOVA with Dunnett post-test vs. ethanol control, N = 6.

As we had observed a decrease in the cell viabilityafter treatment with the an1,25Ds, we examined how the an1,25Ds affected the expression of the proliferation marker Ki67 in the treated cells. However, there were no significant differences in the percentage of Ki67-positive cells, independent of treatment and cell line (Figure 8, Supplementary Table S3 for values normalized to ethanol control). One explanation for the lack of downregulation could be that in these cells p53 is mutated [27], which might impair its ability to downregulate Ki67 [29].

## 3. Discussion

Numerous studies have shown that calcitriol had anticancer effects in different models of cancer [4]. However, a major drawback of its use in therapeutic super-physiological doses is the potentially lethal calcemic effect. This led to the development of analogs with lower calcemic effects, but with similar, or stronger anticancer properties [30,31]. In the present study, we have tested for the first time if single- and/or double-point modified analogs of the most active form of vitamin D are effective in HGSOC cells. We demonstrated that our an1,25Ds were able to induce *CYP24A1* expression, suggesting that the vitamin D signaling system is working in these cells. The analogs also increased nuclear VDR levels and reduced cell number—effects which were time-, structure of the analog- and cell line-dependent.

This pilot study is the first paper testing the effectiveness of analogs of active vitamin D in patient-derived high-grade serous ovarian cancer cell lines. Our purpose was to assess if these analogs are able to induce an effect in these cells, and not to understand the whole picture of the functional profile of these compounds. For this purpose, we used two synthetic analogs of vitamin D modified at a single point, PRI-1906 with an extended and rigidified side chain, and its side-chain C-26-, C-27-homolog, PRI-1907. We have also used two double point-modified analogs, PRI-5201 and PRI-5202, additionally depleted of the 19-methylene in their A-ring (see Figure 1) and thus viewed as 19-*nor* variants of the former two [32]. We have compared their activity with calcitriol, as we already knew that the an1,25Ds, except PRI-1907, were less calcemic in mice than calcitriol [33]. In this study, we proved that the response of HGSOC cell lines depends on the structure of the respective an1,25Ds sensitive and the most pronounced activity was found for the double point-modified analogs (modified A-ring and side-chain). Compared to calcitriol, the most active analog PRI-5202 was a 19-*nor* compound extended at both C-26 and C-27.

The activity of the compounds was cell-line dependent, a property already observed earlier in other cell lines [33]. The differences were most pronounced at low concentration (1 nmol/L), while at high concentration (100 nmol/L), the differences leveled out. PRI-5202 was the most potent analog in inducing *CYP24A1* expression in both HGSOC cell lines, while PRI-1907 was the least potent one. Dual activity of PRI-1907 has been observed previously in HL60 cells, where this compound had the highest pro-differentiating effect, while it was least potent in inducing *CYP24A1* mRNA expression when compared with PRI-1906 and calcitriol [34]. This analog, although one of the most active analogs in leukemia and breast cancer models, had also the highest toxicity in animal models [22]. PRI-5202, the 19-nor version of PRI-1907 (see Figure 1), was much less toxic in animal models and had the lowest calcemic effect compared with all other an1,25Ds [22,33]. In the tested HGSOC cells, PRI-5202 was the most active in increasing *CYP24A1* expression and reducing cell number even in the less responsive 14433 cells. Except for PRI-1907, all other an1,25Ds were more active than calcitriol, when used at 1 nmol/L. This difference has disappeared when the an1,25Ds were used at 100 nmol/L. The effect of PRI-1906 and PRI-5201 was very similar at all concentrations and treatment times.

Although there are some reports on the activity of calcitriol in ovarian cancer cells little is known about the functioning of an1,25Ds in these cells. Previous studies have shown that calcitriol inhibited the expression of genes involved in proliferation, epithelial–to-mesenchymal transition, the Wnt pathway. However, most of these studies were performed in the commercially available cell lines OVCAR3 or SCOV3 [35,36,37], which are less reliable models of the most common type of ovarian cancer, the high-grade serous ovarian cancer. This is why we have used two of our patient-derived cell lines, chosen from a whole panel of HGSOC cells. The two tested ovarian cancer cell lines responded differently to the analogs depending on the duration of the treatment, manifesting in differential up- or downregulation of several target genes.

All an1,25Ds, except PRI-1907, were very potent in inducing *CYP24A1* gene expression, as their EC_50_-s were in the nanomolar range. The potency of the analogs (measured after 4 h treatment) was 2–3 times higher in the 14433 cells than in the 13781 cells; on the other hand, the efficacy of the compounds (increasing *CYP24A1* expression from basal level) was higher in the 13781 line. This difference between the efficacies of the drugs in the two cell lines increased with the duration of the treatment. We suppose that this difference was due to the differences in the basal expression level of *CYP24A1*: in the 14433 cells, with higher basal levels of *CYP24A1*, the effect of the analogs is lower, because the already existent CYP24A1 enzyme degrades the analogs, at least partially. The significant accumulation of nuclear VDR in the 13781 cells treated with the analogs might be another explanation for the higher responsiveness of these cells to the treatment with the an1,25Ds. These cells were also significantly less viable after treatment with the an1,25Ds than the 14433 cells.

In this study, we showed that the single- and double-point modified analogs of 1,25D_2_ are active in HGSOC cells, and their activity depends on the cell-line and the chemical structure of the an1,25Ds. Next, we need to explore whether these compounds have anti-cancer effects in ovarian cancer cells. There is also an urgent need for new an1,25Ds which would be effective also in cell lines like 14433 and to discover what makes some HGSOCs responsive to an1,25Ds and others not. Based on these considerations, our results underline the necessity of testing more patient-derived cell lines, to understand the biological activity of the an1,25Ds in ovarian cancer. Understanding what makes these cells responsive to the analogs could help in designing analogs that have even higher anticancer activity and retain low toxicity.

## 4. Materials and Methods

### 4.1. Compounds

Calcitriol and the analogs (PRI-1906, PRI-1907, PRI-5201, PRI-5202) were obtained and handled as previously described [19,32]. All an1,25Ds were dissolved in ethanol, which was used as vehicle control at a maximum concentration of 0.1%.

### 4.2. Cell Culture

The patient-derived high-grade serous ovarian cancer cell lines (13781 and 14433) were selected from a panel of 34 cell lines [27]. Informed consent was obtained from all patients with HGSOC included in this study in the Department of Obstetrics and Gynecology, Medical University of Vienna. The study protocol was approved by the relevant ethics committees (EK No. 366/2003 and 260/2003). The cells were cultured in DMEM/F-12 (1:1) + GlutaMAX containing 10% fetal calf serum (FCS), 100 U/mL Pen-Strep (all Thermo Fisher Scientific, MA, USA). For the treatments with an1,25Ds, the cells were cultured for 2 days and then washed with phosphate-buffered saline (PBS) and cultured in the same medium containing only 2% FCS for the whole duration of the treatment.

### 4.3. Cell Number & Cell Viability

To measure cell number and viability, the cells were treated with 100 nmol/L of each compound for 5 days. For cell number, the cells were washed with PBS, detached using Trypsin-EDTA (Thermo Fisher Scientific) and the number of cells was determined using an automated cell counter TC10TM (Biorad, CA, USA). Cell viability was measured using CellTiter-Blue ^®^ Cell Viability Assay (Promega, WI, USA) according to the manufacturer’s protocol. Fluorescence was measured in black-well plates from the bottom using a Tecan Infinite M200 PRO (Tecan AG, CH) with the following parameters: excitation 550 nm, emission 600 nm.

### 4.4. RT-qPCR

#### 4.4.1. mRNA Expression Analysis

RNA was isolated using EXTRAzol Reagent (Blirt, PL) according to the manufacturer’s protocol. cDNA was synthesized using a High Capacity cDNA Reverse Transcription Kit (Thermo Fisher Scientific). *CYP24A1* gene expression was analyzed in a quantitative polymerase chain reaction (qPCR) using Power SYBR Green PCR Master Mix (Thermo Fisher Scientific). Primers used for the reactions were *GAPDH*: fwd: 5′-TCCTCTGACTTCAACAGCGAC-3′, rev: 5′-TGCTGTAGCCAAATTCGTTGTC-3′; *CYP24A1*: fwd: 5′-CAAACCGTGGAAGGCTATC-3′, rev: 5′-AGTCTTCCCCTTCCAGGATCA-3′; *CXCL1:* fwd: 5′-GAAAGCTTGCCTCAATCCTG-3′, rev: 5′-CTTCCTCCTCCCTTCTGGT-3′; *CXCL2*: fwd: 5′-GGGCAGAAAGCTTGTCTCAA-3′, rev: 5′-GCTTCCTCCTTCCTTCTGGT-3′; *IL-6*: fwd: 5′-AATTCGGTACATCCTCGACGG-3′, rev: 5′-GGTTGTTTTCTGCCAGTGCC-3′; *IL-8*: fwd: 5′-GTTGGCAGCCTTCCTGATTT-3′, rev: 5′-TTCTTTAGCACTCCTTGGCAAAA-3′. Acquired data were analyzed using the 2^−ΔΔCT^ method, [38] using *GAPDH* as the housekeeping gene and total human RNA (Takara, JP) as calibrator.

#### 4.4.2. Quantitative Assessment of *CYP24A1* Transcript

To measure *CYP24A1* transcripts quantitatively, the following primers were used: fwd: 5′-CTCATGCTAAATACCCAGGTG-3′, rev: 5′-TCGCTGGCAAAACGCGATGGG-3′. The standard curve obtained using the amplicon of a known quantity, with the sequence matching the one produced from cDNA using these primers, was obtained in RT-qPCR. Constitutive expression of *CYP24A1* in 14433 and 13781 cells was measured from the cDNA, and the number of transcripts was read out from the standard curve.

### 4.5. Immunofluorescence

For immunofluorescence staining, cells were cultured on 8-chamber slides (Thermo Fisher Scientific). Cells were treated with 100 nmol/L of each compound for 5 days. After treatment, cells were fixed using 3.6% formaldehyde in PBS for 15 min. The fixed cells were incubated for 20 min in 0.2% Triton-X in PBS at room temperature (RT) for permeabilization followed by incubation with 50 mmol/L NH_4_Cl for 15 min and 30 min in 3% BSA in PBS at RT for blocking. Cells were incubated with primary VDR (1:200) antibody (Merck Darmstadt, DE) for 1 h at RT or Ki67 (1:500) (Thermo Fisher Scientific) antibodies for 1 h at RT. An Alexa Fluor 647 conjugated anti-rabbit secondary antibody was used at a concentration of 1:500 in PBS for VDR staining visualization. Next cells were incubated with Ki67 (1:500) antibody (Thermo Fisher Scientific) for 1 h at RT. The Ki67 antibody had a fluorescence marker. Cells were counterstained using DAPI (Thermo Fisher Scientific) and mounted using Fluoromount G (Southern Biotech, Birmingham, AL, USA). Images of the stained cells were acquired using TissueFAXS hard- and software (TissueGnostics GmbH, Vienna, Austria) equipped with a Zeiss AxioImager Z1 using a Zeiss NeoFluar 20×/0.5 objective (Zeiss, Oberkochen, Germany).

### 4.6. Image Analysis

The acquired images were automatically analyzed using TissueQuest 6.0 software (TissueGnostics GmbH) using individual cell detection based on nuclear segmentation. A nuclear mask was used to detect the levels of Ki67 and VDR. Thresholds were set manually based on visual inspection to discriminate Ki67 positive from Ki67 negative cells and propagated to all samples. For VDR, mean pixel-intensities for each cell were used to determine the average grey value (=staining intensity) for each cell.

### 4.7. Data Analysis

Data analysis was performed using Microsoft Excel (Microsoft, Redmond, WA, USA) and statistical analysis was performed using GraphPad Prism 7.0 (Graphpad Software, San Diego, CA, USA). Employed statistical tests are described in the respective figure legends.

## Figures and Tables

**Table 1:** Increase in *CYP24A1* expression in the 13781 cells relative to the expression increase in the 14433 cells.

13781/14433	1 nmol/L for 4 h ^1^	100 nmol/L for 4 h ^1^	100 nmol/L for 5 Days ^1^
**calcitriol**	1.54	7.48	24.25
**PRI-1906**	2.02	8.50	12.58
**PRI-1907**	0.93	2.50	13.34
**PRI-5201**	1.79	7.97	12.43
**PRI-5202**	5.80	8.49	12.71

^1^ Values are presented as *CYP24A1* expression increase from the basal level (% ethanol control) in 13781 cells as a fraction of the increase in 14433 cells.

## Supplementary Materials

All data are available online at https://www.mdpi.com/article/10.3390/ijms23010172/s1.
