# Supplementary material for: Vitamin D Analogs Regulate the Vitamin D System and Cell Viability in Ovarian Cancer Cells
Source: Int J Mol Sci. 2021 Dec 24;23(1):172. doi: 10.3390/ijms23010172 (PMC8745402; doi:10.3390/ijms23010172)
Supplement: Supplementary file 1 [file ijms-23-00172-s001.zip › ijms-1413220-supplementary.pdf]

Table S1. Increase in CYP24A1 expression in the 13781 cells used to calculate ratiosi n Table 1 of the main manuscript.

13781	1 nmol/l for 4 h[1]	100 nmol/l for 4 h[1]	100 nmol/l for 5 days[1]
	Mean % ± SD	Mean % ± SD	Mean % ± SD
**Calcitriol**	277 ± 146	29,752 ± 14,351	1,143,611 ± 437,431
**PRI-1906**	1,461 ± 1,188	42,447 ± 25,012	1,318,811 ± 90,432
**PRI-1907**	104 ± 35	4,282 ± 667	1,285,482 ± 91,310
**PRI-5201**	1,387 ± 961	28,577 ± 13,838	1,317,940 ± 148,390
**PRI-5202**	9,806 ± 3,586	38,880 ± 20,950	1,237,736 ± 230,694

[1]Values are presented as *CYP24A1* expression increase from the basal level (% ethanol control).

Table S2. Increase in CYP24A1 expression in the 14433 cells used to calculate ratios in Table 1 of the main manuscript.

14433	1 nmol/l for 4 h[1]	100 nmol/l for 4 h[1]	100 nmol/l for 5 days[1]
	Mean % ± SD	Mean % ± SD	Mean % ± SD
**Calcitriol**	180 ± 54	3,976 ± 1,581	47,169 ± 40,888
**PRI-1906**	725 ± 260	4,994 ± 2,076	104,824 ± 43,531
**PRI-1907**	112 ± 32	1,715 ± 655	96,334 ± 34,603
**PRI-5201**	777 ± 474	3,585 ± 1,285	106,062 ± 53,173
**PRI-5202**	1,691 ± 1,022	4,577 ± 2,140	97,410 ± 49,649

[1]Values are presented as *CYP24A1* expression increase from the basal level (% ethanol control).

Table S3. Effect of the compounds on VDR and Ki67 protein levels, relative to ethanol control. In contrast, figure 6 shows the actual mean grey value (= fluorescence intensity) for VDR and figure 8 shows the actual percentage of Ki67 positive cells of all cells.

	**13781**		**14433**	
	**VDR**	**Ki67**	**VDR**	**Ki67**
	Mean % ± SD	Mean % ± SD	Mean % ± SD	Mean % ± SD
**Ethanol**	100 ± 34	100 ± 13	100 ± 23	100 ± 18
**Calcitriol**	120 ± 36	83 ± 30	110 ± 17	100 ± 17
**PRI-1906**	122 ± 38	92 ± 21	111 ± 27	101 ± 15
**PRI-1907**	115 ± 30	90 ± 21	104 ± 14	100 ± 16
**PRI-5201**	125 ± 27	92 ± 21	122 ± 27	104 ± 15
**PRI-5202**	135 ± 46	99 ± 27	114 ± 17	103 ± 16

Values are presented as % mean ethanol control.

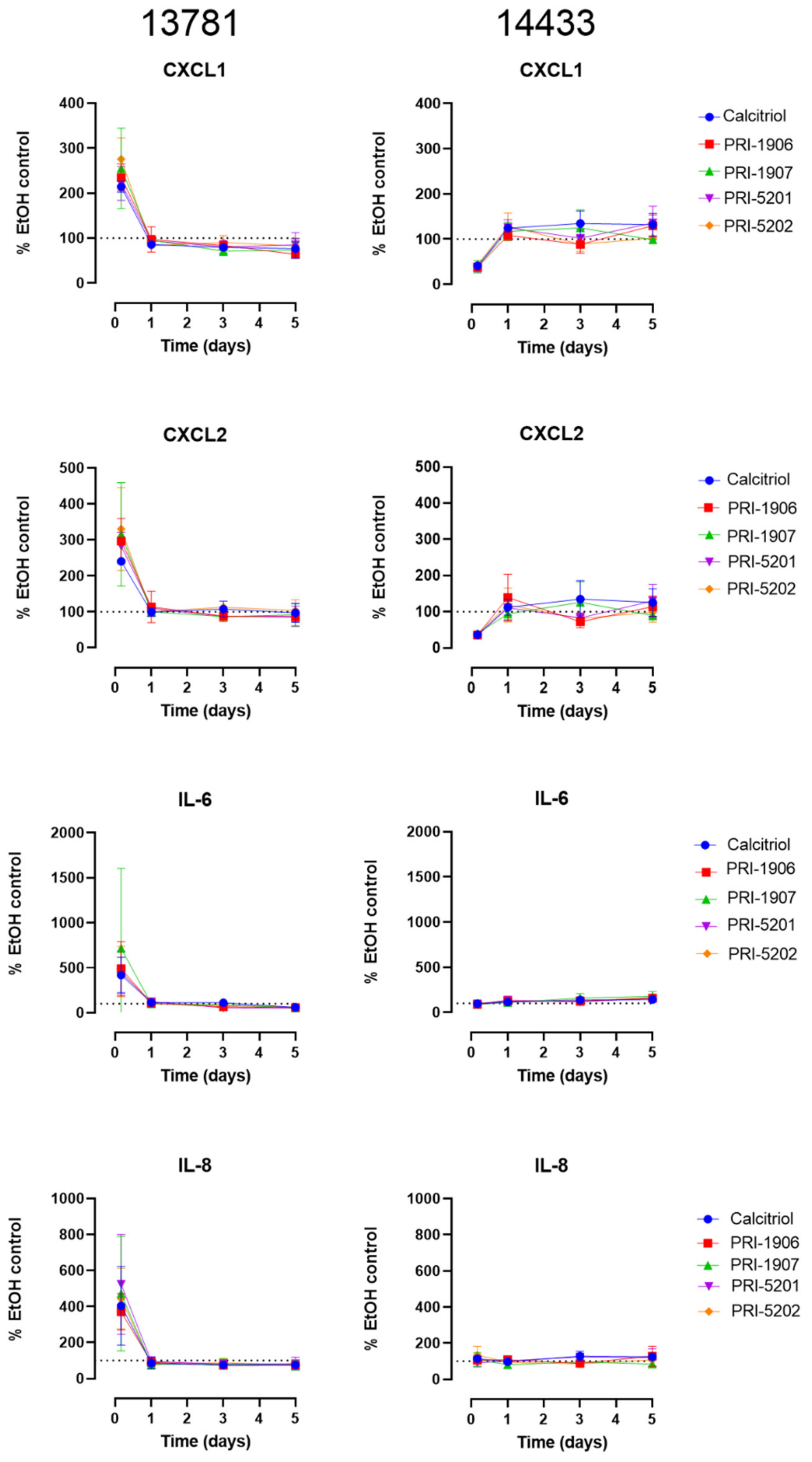

Figure S1. Gene expression in 13781 and 14433 cells after treatment with an1,25Ds at 100 nmol/l at different time points (4 hours, 1 day, 3, and 5 days). The diagrams show the percentage increase compared with the ethanol control. N = 3.
